# Neck-specific exercise for radiating pain and neurological deficits in chronic whiplash, a 1-year follow-up of a randomised clinical trial

**DOI:** 10.1038/s41598-020-62722-4

**Published:** 2020-04-21

**Authors:** Maria Landén Ludvigsson, Gunnel Peterson, Anneli Peolsson

**Affiliations:** 1 Department of Health Medicine and Caring Sciences, Division of Prevention Rehabilitation and Community Medicine, Unit of Physiotherapy, Linköping University, Linköping, Sweden; 2 Rehab Väst, County Council of Östergötland, Department of Health Medicine and Caring Sciences, Linköping University, Linköping, Sweden; 3 Centre for Clinical Research Sörmland, Uppsala University, Uppsala, Sweden

**Keywords:** Pain management, Rehabilitation, Randomized controlled trials

## Abstract

Up to 90% of people with neurological deficits following whiplash injury report chronic symptoms. A recent unique study of neck-specific exercise showed positive results (post-intervention at 12 weeks), regarding arm pain and neurological deficits in people with chronic whiplash associated disorders (WAD). This 1-year follow-up of that randomised controlled study with assessor blinding aimed to examine whether neck-specific exercise with (NSEB) or without (NSE) a behavioural approach has long-term benefits over physical activity prescription (PPA) regarding arm pain and neurological deficits (n = 171). Interventions were: NSE, NSEB, or PPA. Follow-up of arm pain, paraesthesia bothersomeness (questionnaires) and clinical neurological tests were performed after 3, 6 and 12 months and analysed with Linear Mixed Models and General Estimating Equations. The NSE and/or NSEB groups reported significantly less pain and paraesthesia bothersomeness as well as higher odds of normal key muscle arm strength and of normal upper limb neural tension over the year (all p < 0.03), compared with PPA. In conclusion, results suggest that neck-specific exercise with or without a behavioural approach may have persisting long term benefits over PPA regarding arm pain and clinical signs associated with neurological deficits in chronic WAD.

## Introduction

Whiplash-associated disorders (WAD) present a significant public health problem with an incidence of at least 300 per 100,000^1^. After a whiplash injury resulting in neurological deficits (e.g. abnormal reflexes, reduced muscle strength and/or altered sensibility), the recovery rate is low. After 1 year, up to 90% still report pain and other symptoms^2–4^. Individuals with neurological deficits, (WAD grade 3^5^), also suffer more than those without^2,6^. In clinical practice, antidepressants, analgesics/non-steroidal anti-inflammatory drugs and muscle relaxants are often prescribed despite the lack of evidence to support the use of either for radiating pain^7^. Disc protrusions/prolapses may cause neurological deficits in WAD. Furthermore, prolapses seem to progress over time in WAD^8^. A brachial plexus traction injury^9^ may be another cause. A recent MRI-study reports that morphological changes in the brachial plexus and median nerve can be found in people with chronic arm and neck pain following a whiplash injury, even without loss of reflexes or key muscle strength^10^. Protective shoulder elevation, which may reduce brachial nerve tension^11^ and thus also reduce arm pain, is common on the painful side. Altered muscle function^12^ and difficulty in relaxing the trapezius muscle, as detected with electromyography/ultrasound, are reported in WAD^13^. Dysfunction of predominantly the deep cervical muscles in WAD^14–16^ could be another explanation for the increased activity of superficial muscles. Control of intersegmental motion, and thus stability, depends on the deep muscle layers^17^. Ligaments may account for only 25% of the cervical stability^18^, and the deep muscles are thus of great importance in maintaining the vertebrae within the neutral zone^19^ where the loading of supporting structures is optimally distributed^20^. Exercise of these muscles thus seems to be a feasible treatment option. In chronic non-specific neck pain, neck-specific exercise, including the deep muscles, can reduce arm pain^21^. However, both physical and psychosocial factors can be attributed to persistent symptoms in WAD^22^. A behavioural approach may thus be of additional value in the management of chronic WAD, as reported in chronic back pain^23^. Self-directed general physical activity to be performed outside the health care system (physical activity prescription, PPA) is also often recommended for people with chronic musculoskeletal pain^24^.

People with WAD grade 3 are rarely included in treatment studies, and there is thus a lack of evidence of effective treatment of radiating pain in WAD. To our knowledge, only one randomised study has included and evaluated the effect of treatment for radiating pain and signs associated with neurological deficits in chronic WAD^25^. In this study, from our research group, improvements were seen immediately following 12 weeks of neck-specific exercise regarding both radiating pain and signs associated with neurological deficits. However, no improvements were seen following PPA (24). The long-term effects are however unknown.

The aim of this current analysis was to examine whether neck-specific exercise with or without a behavioural approach also has long-term benefits over PPA regarding clinical signs and arm pain associated with neurological deficits in people with chronic WAD.

## Materials and Methods

### Design, procedure and participants

This is a secondary analysis of a randomised controlled multi-centre study with a 1-year follow-up of people experiencing chronic WAD grade 2 or 3 for 6–36 months performed in 2011-2013^26^. Inclusion criteria for the main study also included a Neck Disability Index score (NDI)^27^ of >10/50 points, and/or an average neck pain intensity of >20/100 mm on the visual analogue scale (VAS) relating to pain experienced in the week prior to reporting the VAS. Exclusion criteria included myelopathy, spinal infection, tumours/malignant disease, previous cervical surgery, direct head trauma, more dominant pain elsewhere, earlier neck trauma with persistent neck problems, neck pain causing more than 1 month´s work absence the year before the trauma, diseases or other injuries that might prevent full participation in any of the interventions, severe psychiatric disorder, drug abuse or insufficient knowledge of the Swedish language^28^.

In the current analyses, only participants reporting arm pain without other known causes, and with alterations in either sensibility and/or key muscle strength and/or reflexes were included in the analysis (n = 171). The mean age of participants was 40 years (SD 11, range 18–63). There were 112 (65%) females and 59 (35%) males (Table 1).

**Table 1:** Baseline variables.

	NSE (n = 59)	NSEB (n = 59)	PPA (n = 53)	P-value
Age, mean (SD)	38 (11)	41 (12)	42 (11)	0.10
Gender, female, % (n)	71 (42)	69 (41)	55 (29)	0.14
Months since injury, mean (SD)	19 (8)	20 (9)	20 (11)	0.62
Use of analgesic drugs yes (%)*	51 (30)	64 (38)	70 (37)	0.10
Smoker, yes % (n)	27 (16)	12 (7)	17 (9)	0.11
Educational level % (n)				0.64
*Edu level, primary/secondary school*	7 (4)	8 (5)	11 (6)	
*Edu level, upper secondary school*	56 (33)	56 (33)	55 (29)	
*Edu level, university*	34 (20)	30 (18)	30 (16)	
*Edu level, other*	3 (2)	5 (3)	2 (1)	
Employed	75 (44)	76 (45)	67 (36)	0.79
Neck pain VAS, med (IQR)	38 (21–64)	50 (24–68)	53 (25–61)	0.63
Neck Disability Index, mean (SD)	17 (6)	18 (7)	18 (7)	0.49
Positive prov. test % (n)	35 (20)	39 (21)	47 (23)	0.45

NSE = Neck-specific exercise group, NSEB = Neck-specific exercise group with a behavioural approach, PPA Prescription of physical activity group, WAD = whiplash associated disorder, VAS = Visual Analogue Scale 0–100, Neck Disability Index 0–50, Prov.test = Positive Spurling’s and/or neck traction test, med = median, IQR = inter quartile range *Analgesics/NSAID/antidepressants/muscle relaxants, and one participant took gabapentin. Edu = educational, n = 171.

Participants were recruited between 2011 and 2012 based on medical records, with 1 year follow-ups until 2013, they also underwent a telephone interview and physical examination^28^. Following informed consent, allocation was via a computer-generated list, handled by an independent researcher who placed the results in opaque envelopes for further distribution to the treating physiotherapists. The study, conducted in accordance with the Declaration of Helsinki, was approved by the Regional Ethics Committee of Linköping University, Sweden. The research is reported in accordance with the CONSORT guidelines.

### Interventions and settings

All three interventions took place over a 12-week period, and all participants were advised to continue exercising in accordance with their intervention, and to be generally physically active post-intervention. Treating physiotherapists working in primary care settings in 6 counties of Sweden were selected and matched to one of the three interventions, to work within their field of interest and knowledge as far as possible. The treating therapists had at least 2 years’ experience (2–41 years) of working with musculoskeletal disorders. They also participated in a 1-day workshop and were provided with standardised verbal and written information, including checklists, and were given the opportunity to practise their interventions. Check lists were collected post-intervention, and the physiotherapists were urged to contact the project leaders whenever needed. The timeframe and specific components of the interventions have been previously presented^28^ but are presented briefly below.

### Neck-specific exercise (NSE)

Twice weekly, neck-specific exercise focusing on the deep cervical muscles was performed with a physiotherapist, in addition to home exercises. After initial gentle activation of the deep muscles in lying and sitting, gym exercises mostly using a weighted pulley were introduced. Repetitions without pain provocation were progressively increased, focusing on good posture and low load endurance. A detailed description of the exercises can be found at the Academic Archive On-line^29^

### Neck-specific exercise with a behavioural approach (NSEB)

In accordance with the concept of graded exercise, participants were encouraged to ignore any temporary increase in neck pain and to focus on success in exercise progression^30^ with the same exercises as those undertaken by the NSE group. However, participants were instructed to avoid provocation of radiating arm pain. Participants also received behavioural interventions including education and introduction to activities aimed at pain management (e.g., relaxation, breathing exercises) and problem-solving^28^.

### Prescription of physical activity (PPA)

Participants were prescribed individualised general physical activity, (e.g., Nordic walking, gym classes, individual home exercises that were not neck-specific) to be performed outside the health care system. The chosen activities were based on a short motivational interview and a subsequent physical examination. One follow-up visit or telephone call was encouraged.

### Data collection and outcomes

The data, collected at baseline and at 3, 6 and 12 months, were reported by six independent researchers (experienced physiotherapists) who also conducted the tests and were blinded to intervention type. The 3-month results have already been reported^28^, but are included in the current analyses regarding effect over time.

### Arm pain and paraesthesia

Arm pain, the primary outcome in these analyses, was measured as current arm pain, maximum and minimum level of arm pain experienced in the preceding week on a VAS scale (0 = no pain, 100 = worst imaginable pain). The percentage of participants with a pain reduction of at least 50%, indicating substantial improvement, is also reported, as recommended by The Initiative on Methods, Measurement and Pain Assessment in Clinical Trials (IMMPACT)^31^. Minimum arm pain was also used to evaluate whether the pain was constant or intermittent since it can be an important prognostic factor for pain relief^32^. A minimum level of <3 mm on the VAS was regarded as no pain^33^.

Secondary outcomes were arm paraesthesia bothersomeness in the preceding 24 hours (VAS, 0 = not bothersome at all, 100 = extremely bothersome). Frequency of arm pain and of paraesthesia were recorded with a five-point scale from never to constantly, as previously used in studies of cervical radiculopathy^34^.

Adherence to the prescribed post-intervention exercise was estimated by the participant as part of a questionnaire on a 4-point scale. The question was: Have you continued with the exercise that your physiotherapist in this study recommended during the last 3 months (or during the last 6 months, which was asked at the one year follow-up) The answers were: Yes, I have exercised completely according to plan, or, more than half of what was planned, or, less than what was planned, or No, I have not continued exercising at all. Exercise diaries were also collected until the 6-month follow-up. The participants who had continued exercising as planned by at least 50% were regarded as adherent.

### Clinical outcomes

All clinical tests (secondary outcomes) were performed by female physiotherapists, blinded to the interventions, with an average of 19 years of clinical experience of assessing and treating musculoskeletal disorders. The test order was standardised and in the same order as the outcomes are presented below.

Sensibility was tested with a pinprick wheel and a soft brush at the following locations: supraclavicular space (C4), lateral upper arm below the deltoid (C5), thumb (C6), 3^rd^ digit (C7), and 5^th^ digit (C8). Responses were classified as normal or abnormal (hypo-, hyper- or dysesthesia, or allodynia). Key muscle strength of the deltoids, biceps, triceps, wrist extensors, wrist flexors, finger flexors, and finger abductors was tested manually, and classified as normal or decreased (from 0 = no contraction to 5 = normal strength). Deep tendon reflexes (biceps, brachioradial, triceps) were classified as normal or abnormal (hypo- or hyperreflexia, or areflexia)^35^. The Upper Limb Neural Tension test (ULNT) with median nerve bias (ULNT-A) was used to evaluate neural pathology by stressing nervous tissue^36^. The ULNT test was positive (provocative) when reproducing familiar arm pain and protective muscle tension occurred.

Data can be shared upon reasonable request.

### Statistics

The sample-size calculation was based on the primary outcome in the main study, the NDI score (3.5/50, SD7, alpha 5%, power 80%).

Descriptive statistics were calculated, and between-group comparisons at baseline were evaluated with the Kruskal-Wallis test for non-parametric data, with the Mann-Whitney U test for post-hoc testing, or with one-way ANOVA for normally distributed parametric data. For dichotomous outcomes, chi-square tests were used. For two-group comparisons, such as drop-out analysis, independent samples t-tests, chi-square tests or Mann-Whitney U test as appropriate were used.

The main analyses were made on an intention-to-treat basis, including all available data at each time point. Linear Mixed Models (LMM) were used to analyse the normally distributed VAS change-scores at the three group levels. A factor analytic, first order heterogeneous covariance matrix was used. For dichotomous and ordinal outcomes Generalised Estimating Equations (GEE), with a binary or ordinal logistic model and unstructured covariance matrix, were used which rendered results in odds ratios (OR). For both the GEE and LMM models, overall time (all groups together), group (general mean difference between intervention groups), and a group-by-time interaction were included as fixed factors. Both methods include all available time points for each participant. The significance level was set at p ≤ 0.05 for all analyses and corrected to allow for multiple post-hoc tests using a Bonferroni adjustment.

For a sub-analysis of a proportion of participants with at least 50% reduction of pain/bothersomeness, a chi-square test was used, and participants who reported less than 3 mm on the variable in question (= no pain^33^) at baseline were not included. SPSS version 23 was used (SPSS Inc, Chicago, IL, USA).

## Results

**Figure 1:**
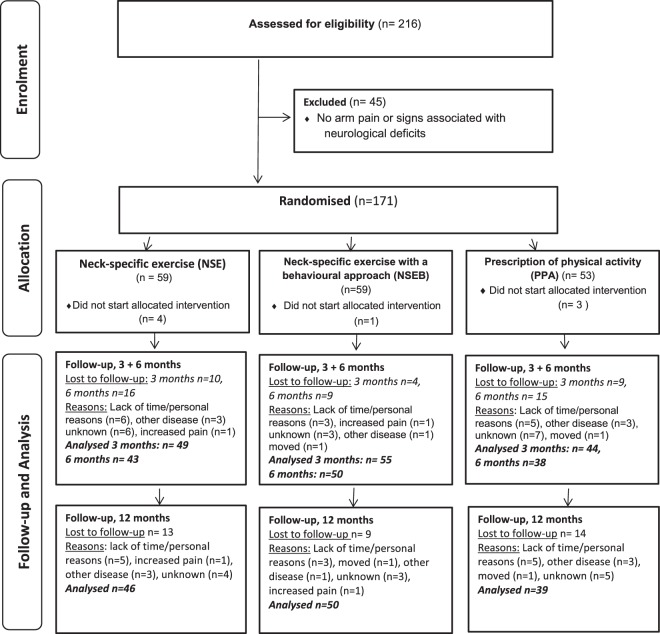
Flow chart of participants.

There were no differences between allocation groups in any variables at baseline (Table 1). The 1-year follow-up was completed by 79% of participants (n = 135). There was no difference between dropouts and completers regarding gender, age or any of the outcomes (all p > 0.10). The number analysed at each time point can be seen in Fig. 1.

### Pain/paraesthesia bothersomeness

**Figure 2:**
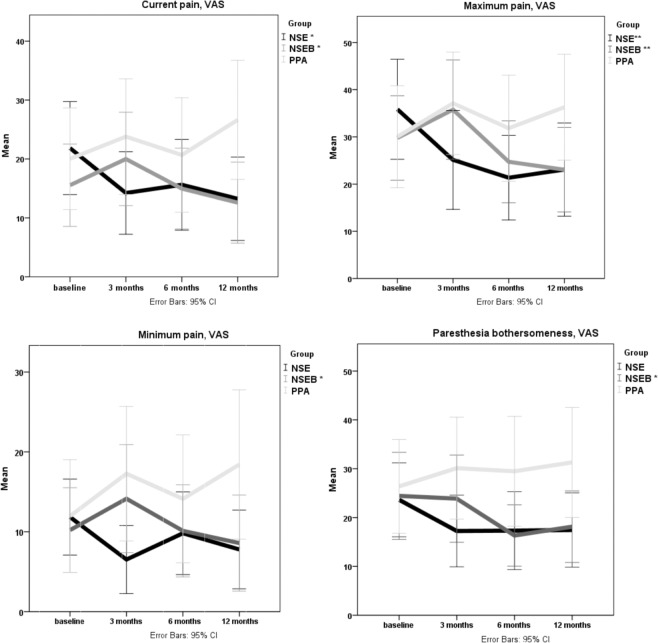
Mean pain and paraesthesia bothersomeness VAS-scores NSE = Neck-specific exercise, NSEB = Neck-specific exercise with a behavioural approach, PPA = Prescription of physical activity, *p < 0.05 within group **p < 0.01.

There were significant main group effects on all primary outcomes (current, minimum, maximum pain p < 0.01) and paraesthesia bothersomeness (p = 0.03) (Table 2). The post-hoc tests revealed significant improvements for the NSE group compared with the PPA group in all pain outcomes and in bothersomeness (p < 0.01 to 0.05) except odds of reduced pain frequency (p = 0.09). The NSEB group was improved compared with the PPA group regarding current pain (p = 0.02) and no minimum arm pain (p < 0.01). There were no differences between the NSE/NSEB-groups, and there were no improvements in any of the outcomes for the PPA-group, which tended to deteriorate over time (Tables 2–4). As seen in Fig. 2 the NSEB group was significantly improved over time in all four outcomes measured with VAS and the NSE group was improved in two outcomes (p < 0.01 to <0.05). The time and interaction effects including all groups were insignificant except in pain frequency, where the interaction was significant. This indicates that the changes remained over time.

**Table 2:** Change scores of pain and paraesthesia bothersomeness over time.

	NSE						NSEB						PPA						P-values		
	Change scores, from baseline to:						Change scores from baseline to:						Change scores from baseline to:						Group	Time	Inter-action
	3 months	95% CI	6 months	95% CI	12 months	95% CI	3 months	95% CI	6 months	95% CI	12 months	95% CI	3 months	95% CI	6 months	95% CI	12 months	95% CI			
Current arm pain VAS, mean (SD)	8 (25)	0–16	6 (23)	−1–13	9 (19)	2–15	−4 (16)	−9–0	1 (13)	−3–4	3 (19)	−3–9	−4 (27)	−13–6	−1 (21)	−8–7	−7 (21)	−14–1	<0.01*	0.83	0.15
Arm pain, maximum, VAS, mean (SD)	11 (27)	2–19	15 (25)	6–23	13 (29)	3–22	−6 (21)	−13–1	5 (24)	−2–12	7 (22)	0–14	−7 (32)	−18–4	−2 (26)	−11–7	−6 (19)	−13–1	<0.01*	0.13	0.13
Arm pain, minimum, VAS, mean (SD)	5 (13)	1–9	2 (15)	−3–7	4 (14)	−1–7	−4 (13)	−8–0	0 (13)	−4–4	2 (13)	−2–6	−5 (22)	−13–3	−2 (14)	−7–3	−6 (16)	−12–1	<0.001*	0.88	0.18
Arm bother-someness, VAS, mean (SD)	6 (26)	−2–15	6 (29)	−3–16	6 (29)	−3–16	1 (22)	−6–7	8 (24)	1–15	6 (25)	−1–14	−4 (25)	−12–5	−3 (29)	−13–7	−5 (23)	−13–3	0.03*	0.30	0.22

All change scores are compared with the baseline value. Positive values denote improvement. NSE = Neck-specific exercise, NSEB = Neck-specific exercise with a behavioural approach, PPA = Prescription of physical activity, CI = confidence interval, VAS = Visual Analogue Scale 0-100 mm, SD = Standard deviation. Results were calculated with linear mixed models. *Bonferroni-post-hoc calculations significant values between groups: NSE-PPA: all pain values p < 0.01, bothersomeness p = 0.03, NSEB-PPA current arm pain p = 0.02, NSE-NSEB no differences. Analysed with LMM, all participants included (n = 171).

**Table 3:** Frequency of pain/paraesthesia bothersomeness and proportion of participants with no clinical signs.

	NSE				NSEB				PPA			
	Percentages (n)				Percentages (n)				Percentages (n)			
	Baseline	3 months^a^	6 months	12 months	Baseline	3 months^a^	6 months	12 months	Baseline	3 months^a^	6 months	12 months
No minimum arm pain	44 (26)	69 (34)	62 (28)	61 (28)	44 (26)	36 (20)	51 (24)	65 (32)	52 (27)	52 (23)	53 (20)	33 (13)
**Frequency arm pain**												
*Occasionally or less*	65 (36)	82 (40)	84 (36)	76 (34)	76 (42)	73 (39)	80 (37)	75 (36)	79 (41)	69 (29)	63 (24)	68 (26)
*Daily/constantly*	35 (19)	18 (9)	16 (7)	24 (11)	24 (16)	27 (14)	20 (11)	25 (12)	21 (11)	31 (15)	37 (14)	32 (12)
**Frequency paraesthesia**												
*Occasionally or less*	73 (41)	84 (42)	77 (34)	77 (34)	71 (41)	72 (39)	85 (40)	76 (37)	69 (36)	66 (29)	58 (22)	68 (26)
*Daily/constantly*	27 (15)	16 (8)	23 (10)	23 (10)	29 (17)	28 (15)	15 (7)	24 (12)	31 (16)	34 (15)	42 (16)	32 (12)
Sensibility, normal	13 (8)	33 (16)	23 (10)	35 (15)	5 (3)	24 (13)	30 (15)	37 (18)	11 (6)	25 (11)	16 (6)	16 (6)
Muscle strength, normal	54 (32)	74 (35)	65 (28)	70 (30)	51 (30)	63 (35)	52 (26)	69 (34)	38 (20)	34 (18)	47 (18)	46 (17)
Tendon reflexes, normal	61 (36)	76 (37)	77 (33)	73 (29)	63 (37)	82 (45)	78 (39)	81 (39)	72 (38)	59 (26)	81 (31)	78 (25)
ULNT-A, non-provocative	55 (31)	56 (24)	35 (15)	74 (31)	43 (25)	56 (26)	32 (16)	75 (36)	57 (30)	37 (15)	45 (17)	51 (19)

^a^ = 3-month data previously presented^25^, NSE = Neck-specific exercise, NSEB = Neck-specific exercise with a behavioural approach, PPA = Prescription of physical activity. ULNT = Upper limb neural tension test. n baseline = 171, n at 3 months = 148, n at 6 months = 131, n at 12 months = 135.

**Table 4:** Clinical signs and frequency of arm pain/paraesthesia bothersomeness, Odds ratios from baseline to 12 months.

	NSE to PPA			NSEB to PPA			NSE to NSEB			Group	Time	Interaction
	B (Std error)	OR (95%CI)	P-value	B (Std error)	OR (95%CI)	P-value	B (Std error)	OR (95%CI)	P-value			
Strength, normal	1.2 (0.5)	0.3 (0.1–0.7)	<0.01*	1.2 (0.5)	0.3 (0.1–0.7)	<0.01*	0.3 (0.3)	0.8 (0.4–1.4)	0.42	<0.01*	<0.01^a^	0.5
Sensibility, normal	0.8 (0.5)	0.4 (0.2–1.2)	0.09	0.9 (0.5)	0.4 (0.2–1.0)	0.06	0.1 (0.4)	1.1 (0.5–2.5)	0.81	0.45	<0.01^a^	0.04*
Reflexes, normal	0.04 (0.3)	1.0 (0.6–1.9)	0.89	0.2 (0.3)	0.8 (0.5–1.5)	0.56	0.2 (0.3)	1.2 (0.7–2.2)	0.53	0.74	<0.01^b^	0.13
ULNT-A nonprov	0.5 (0.3)	1.6 (0.9–2.8)	0.09	0.7 (0.3)	2.0 (1.1-3-7)	0.02*	−0.2 (0.3)	0.8 (0.5–1.3)	0.38	0.06	0.01^a^	0.38
Freq arm pain	0.6 (0.4)	1.8 (0.9–3.7)	0.09	0.2 (0.3)	1.2 (0.6–2.3)	0.56	0.4 (0.4)	1.5 (0.8–2.9)	0.25	0.41	0.92	<0.01*
Freq Bothers	0.7 (0.3)	0.5 (0.3–1.0)	0.05*	0.5 (0.3)	0.6 (0.3–1.2)	0.16	−0.1 (0.4)	0.9 (0.4–1.8)	0.71	0.26	0.68	0.09
No min arm pain	1.1 (0.5)	3.1 (1.3–7.6)	0.01*	1.3 (0.5)	3.8 (1.5–9.2)	<0.01*	−0.2 (0.43)	0.8 (0.4–1.9)	0.65	0.27	0.51	<0.01*

*Denotes significant values, ^a^ = significant at 6 and 12 months, ^b^ = significant at 12 months only, B = unstandardized Beta, OR = Odds ratios, CI = Confidence Interval, NSE = Neck-specific exercise, NSEB = Neck-specific exercise with a behavioural approach, PPA = Prescription of physical activity, ULNT = Upper limb neural tension test, nonprov = non-provocative, Freq= frequency, Bothers= bothersomeness, min= minimum. Analysed with GEE. All participants included, n = 171.

**Figure 3:**
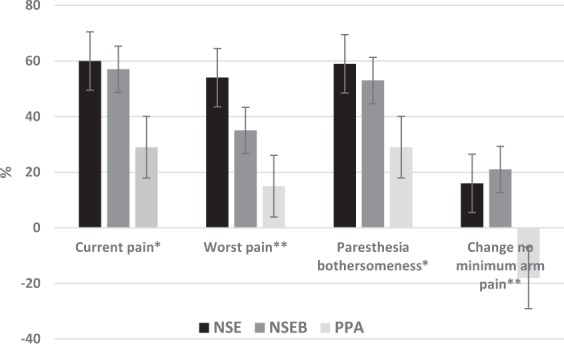
Percentage of participants with at least 50% reduction in arm pain/paraesthesia bothersomeness and change of percentage of participants with no minimum arm pain after 1 year. NSE = Neck-specific exercise, NSEB = Neck-specific exercise with a behavioural approach, PPA = Prescription of Physical Activity *p < 0.05, **p < 0.01. Bars represent standard errors. Number of participants/total with>50% improvement of current pain: NSE 18/30, NSEB 17/30, PPA 7/24, worst pain: NSE 17/31, NSEB 13/37, PPA 4/27, paresthesia NSE 19/32, NSEB 20/41, PPA 10/35. No minimum arm pain indicates <3 mm VAS. Change no minimum arm pain indicates that 16% *more* participants in the NSE group and 21% in the NSEB group, and 18% *fewer* participants reported no minimum arm pain as compared with baseline.

When evaluating the percentage of participants whose pain improved by at least 50%, there were also significant differences favouring the neck-specific groups on all outcomes (p < 0.05). The NSE group had the highest proportion of improved patients regarding pain and bothersomeness. This was also the case with the proportion of participants who reported no minimum pain (p < 0.01). The proportion of pain-free participants in the PPA group had decreased by 18% as compared with the NSE/NSEB groups which reported 16% (NSE) and 21% (NSEB) more participants with no minimum pain at the 1-year follow-up. (Fig. 3).

### Clinical tests and adherence

The odds ratio for improved key muscle strength was significantly higher for the NSE/NSEB groups compared with the PPA group (both ORs 0.3, p < 0.01). The NSEB group also had higher OR regarding a non-provocative ULNT test (OR 2.0, p = 0.02). There was no significant difference in odds between groups regarding reflexes (p > 0.56) or sensibility (p = NSE 0.09, NSEB 0.06). There was no difference between the NSE and NSEB groups for any of the outcomes (Tables 3 and 4).

Adherence at the 1-year follow-up did not differ between groups (p = 0.17, NSE 24%, NSEB 31%, PPA 44%). There was no difference between those who continued exercising post-intervention and those who did not regarding pain/paraesthesia bothersomeness or clinical tests for any of the groups (all p > 0.07). No serious side-effects were registered.

## Discussion

To the best of our knowledge this is the first treatment study to evaluate the effect on arm pain and clinical signs associated with neurological deficits associated with chronic WAD. The NSE group improved over time compared with the PPA group on all main outcomes (current, maximum and minimum arm pain) and also on paraesthesia bothersomeness (frequency and VAS rating) (Tables 2 and 4).

As previously presented^25^ and as seen in Fig. 2, the NSE group was the only group which improved immediately following the intervention. Even though the NSEB group tended to have an increase in pain immediately post-intervention, there was an overall significant improvement over time for current pain and percentage of participants with no minimum arm pain compared with the PPA group. The trend was similar also for the other pain/paraesthesia bothersomeness outcomes in the NSEB group (Fig. 2). The reason for this change post-intervention is not clear but the NSEB intervention allowed for temporary increase of neck pain (albeit not arm pain), and focus was on exercise progress rather than pain. It can be speculated that post-intervention, participants may have focused more on avoiding pain provocation. It is also reasonable to assume that the effect may have been delayed for this group, since they also had behavioural tasks to focus on. Furthermore, the behavioural approach was not directed at radiating nerve pain which may also have an impact on the lack of additional value of NESB. Both NSE/NSEB groups had significant within-group improvements and both groups had similar levels of pain and bothersomeness after 12 months.

The NSE/NSEB groups also had a higher percentage of participants with at least 50% reduction of both pain and paraesthesia bothersomeness, classified as substantial improvement or treatment success by IMMPACT^37^. When analysing this improved group further, the mean improvement was considerably higher than the cut-off, with improvement means of 83–85%. Furthermore, the percentage of participants with no minimum arm pain had increased by around 20% in the NSE/NSEB groups, whereas the PPA-group had an 18% decrease in participants with no minimum arm pain at 12 months compared with baseline. Even though some individuals in the PPA group also improved, it is unclear why there was a general trend for worse pain/bothersomeness. It could be the lack of neck-specific exercise or the lack of guidance. Nevertheless, this is consistent with other outcomes from the main study, such as general health-related quality of life^38^, neck disability^39^, self-reported work ability^40^, pain catastrophising, anxiety and kinesiophobia^41^, where there was also a trend for the PPA group towards deterioration over time. However, as seen in Fig. 2, the lines changed directions at different time points, and part of the explanation may be a small natural variation of symptoms over time even among people with chronic conditions, where no spontaneous changes are to be expected after 3–6 months^5^. Our findings are consistent with a randomised study of females with non-specific neck and arm pain, where a multimodal programme including neck-specific exercise was significantly better at reducing arm pain than advice on aerobic exercise and stretching after 12 months^21^. A recent metanalysis of exercise and cervical radiculopathy concludes that carefully selected exercise can have a good effect on radiculopathy but large-scale studies are needed^42^.

The clinical relevance of our results should also be considered. There are, to our knowledge, no exercise studies which have established the minimal clinically important difference (MCID) values for arm pain in chronic WAD, nor for arm pain associated with general neck pain. The MCID depends on both the clinical condition of the group of patients and the intervention^43^. Such values may therefore be of interest to establish in future studies. In heterogenous populations like WAD a wide variance of change scores can follow specific interventions. Severity of neurological deficits is not homogenous, and a larger variance of response to treatment (change score) could thus be expected. Neck-specific exercise is not a universal cure for chronic WAD. But as seen in Fig. 3, up to 60% of the participants reported at least 50% reduction of pain, and almost 20% reported having completely pain-free moments. This indicates that NSE/NSEB could be important to consider in the management of WAD with associated arm pain.

Regarding clinical outcomes the NSE/NSEB groups demonstrated larger improvements in arm strength directly post-intervention compared with the PPA group^25^, which were maintained over the following year. The odds of having normal arm muscle strength were significantly higher for both neck-specific groups, despite there being no arm exercises in these interventions, as opposed to the PPA interventions where these often were included. Regarding a non-provocative ULNT test there was no difference between groups at 3 months as previously reported^25^, however this changed over time. The odds over time were significantly better for the NSEB group compared with the PPA group, whereas similar odds for the NSE group versus PPA did not quite reach significance. Although the proportion of participants with normal sensibility increased by almost three times in the NSE group, and seven times in the NSEB group, the odds of normal sensibility did not quite reach a significant difference between groups.

As opposed to medication, neck-specific exercise seems to be free from side-effects when performed in accordance with the protocols of this study. It may therefore be an important alternative to pain medication, which aim to reduce pain, but not to work with the muscle alterations reported in chronic WAD.

There was no difference over time between the NSE/NSEB groups in any of the outcomes, neither regarding pain nor clinical signs. This indicates that either of the neck-specific interventions can be tried in people with chronic WAD with arm pain and signs associated with neurological deficits. However, as demonstrated, the decrease in pain was achieved sooner in the NSE group and NSE is also cost-effective compared with both NSEB and PPA^44^. Therefore, NSE may be the first-line choice.

A possible explanation for the good results of NSE could be that keeping the spinal vertebrae in a position where the disc pressure is evenly distributed and/or the intervertebral foramina are not narrowed, could reduce the risk of painful structural nerve pressure and disc herniation. Without the deep muscle activation, important for segmental control^17^, there may be more tension on the ligaments, which may already be elongated following whiplash trauma*.* Increased segmental motion in females with chronic WAD has been reported^45^. And for instance, disc protrusions (which are shown to be more common in WAD) reduce the distance between vertebrae, thus slacking the ligaments which also increases the risk of abnormal glides where there may be increased nerve pressure and thus arm pain. However, this explanation is hypothetical and needs to be evaluated further in future studies.

While the primary outcomes were analysed with LMM as planned, it is not designed to analyse dichotomous outcomes over time, and mean values of such outcomes would be of no clinical relevance. Thus, GEE was chosen, as it is appropriate for dichotomous outcomes (here clinical tests and frequency of pain/bothersomeness) but result in OR instead. Even though the outcomes are thus presented differently, they both demonstrate differences between groups.

Time effects are based on mean changes over time for all groups together. They were insignificant which is not surprising as the groups changed in different directions. However as seen in Fig. 2 this does not mean that there are no within-group changes over time.

When interpreting the results there are some limitations that need to be considered. There was a clear trend towards differences between groups for all outcomes except deep tendon reflexes, but many differences did not quite reach significance. This suggests a somewhat larger sample would have been needed to gain sufficient power for this three-group comparison. The sample size was based on the main outcome of the main study, the NDI.

Another limitation relates to the clinical tests, which are the most commonly used physical tests of neurological deficits in clinical practice^46^. This limitation is naturally also shared with other studies reporting such outcomes which are also the outcomes proposed by the Quebec Task Force for WAD grading^5^. They include tests of key muscles, tendon reflexes, and testing of sensory deficits. However, there is a lack of studies assessing the diagnostic accuracy of these tests^47^. Wainner *et al*. report that most tests have at least a fair level of reliability, and the ULNT-A, also included in our study, is reported to have excellent reliability. The ULNT-A also has very high sensitivity, while the other tests generally have high specificity, but not very high sensitivity^48^. To enhance reliability in our study, the tests were performed by experienced physiotherapists who practised the standardised tests together. Additionally, the individual follow-ups were generally performed by the same physiotherapist who was blinded to the intervention at all time points.

No tests of central sensitisation were done. However hyposensitivity is not a feature of central sensitisation^49^, and it was twice as common as hypersensitivity. Nonetheless it can not be ruled out that some participants may have been less likely to improve in sensibility due to central sensitisation.

Furthermore, neurological deficits were not evaluated spinal segments C4 and above, which is an important challenge for future studies to consider. Compared to people with chronic insidious neck pain, people with chronic WAD more often report pain in the upper part of the cervical spine^50^. The lack of specific deficits in this area is a challenge^51^.

With a large number of treating physiotherapists (n = 58) of both genders, various age and experience across the randomized treatment arms, it is unlikely that these or any other demographic factors of the treating physiotherapist had an impact on the results. The risk of treatment contamination was low due to the interventions being performed at many different clinics, often at different timepoints and led by intervention-specific physiotherapists. Results from such a multi-centre study may also be more generalizable to physiotherapy in primary care however it does offer less control of the performance of the intended interventions.

In conclusion, the findings of this 1-year follow-up study suggest that the positive results of neck-specific exercise on arm pain and signs associated with neurological deficits persist over time in people with chronic WAD. Whether a behavioural approach was also part of the intervention or not, did not have any significant impact on the long-term results, however pain reduction was reported earlier for the group undertaking neck-specific exercise only.
